# Multi-omics analysis reveals that Danggui Buxue decoction ameliorates benzene-induced blood deficiency syndrome via the restoration of hematopoietic lineage and remodeling of the immune microenvironment

**DOI:** 10.1186/s13020-026-01365-5

**Published:** 2026-05-22

**Authors:** Hongda Liu, Junling Ren, Yu Hu, Yu Yang, Hui Sun, Heng Fang, Guangli Yan, Ying Han, Xijun Wang

**Affiliations:** 1 State Key Laboratory of Integration and Innovation of Classical Formula and Modern Chinese Medicines, National Chinmedomics Research Center, National TCM Key Laboratory of Serum Pharmacochemistry, Metabolomics Laboratory, Department of Pharmaceutical Analysis, Heilongjiang University of Chinese Medicine, Heping Road 24, Harbin, 150040 China; 2 State Key Laboratory of Quality Research in Chinese Medicine, Macau University of Science and Technology, Avenida Wai Long, Taipa, Macau China

**Keywords:** Danggui Buxue Decoction, Blood deficiency syndrome, Hematopoietic cell lineage, MHC class II gene cluster, Proteomic, Transcriptomic

## Abstract

Blood deficiency syndrome (BDS) is a systemic disorder characterized by hematopoietic dysfunction and immune dysregulation. Given the limitations of current therapies, such as single efficacy and adverse effects, there is an urgent need for multitarget therapeutic agents with systemic regulatory effects. In this study, a mouse model of BDS was established through chronic benzene inhalation. Using an integrated transcriptomics and proteomics approach, we systematically investigated the therapeutic mechanism of Danggui Buxue Decoction (DBD). The results demonstrated that DBD significantly restored body weight, thymic and splenic indices, and bone marrow microstructure in model mice but also improved peripheral blood parameters such as the red blood cell count and mean corpuscular volume. Furthermore, DBD coordinately modulated serum hematopoietic factor and inflammatory cytokine levels. Mechanistically, DBD exerts its therapeutic effects through dual pathways. On the one hand, it promotes hematopoietic repair by upregulating transferrin receptor (TFRC) to support iron-dependent erythropoiesis, modulating KITLG/FLT3LG to maintain stem cell pool stability, and reprogramming integrin expression (e.g., upregulating ITGA4 and downregulating ITGA1) to facilitate stem cell homing and suppress fibrosis. On the other hand, it reshapes the immune microenvironment by enhancing MHC class II antigen presentation (e.g., H2Aa, H2-Ab1, H2-DMb1, and H2-Eb1) and immune cell activation (e.g., CD22, CD37, CD20, and CD8a), thereby reestablishing immune homeostasis. This study provides a systematic molecular basis for the multitarget and holistic regulatory properties of DBD, supporting its clinical application and suggesting potential therapeutic targets for BDS-related disorders.

**Graphical Abstract:**

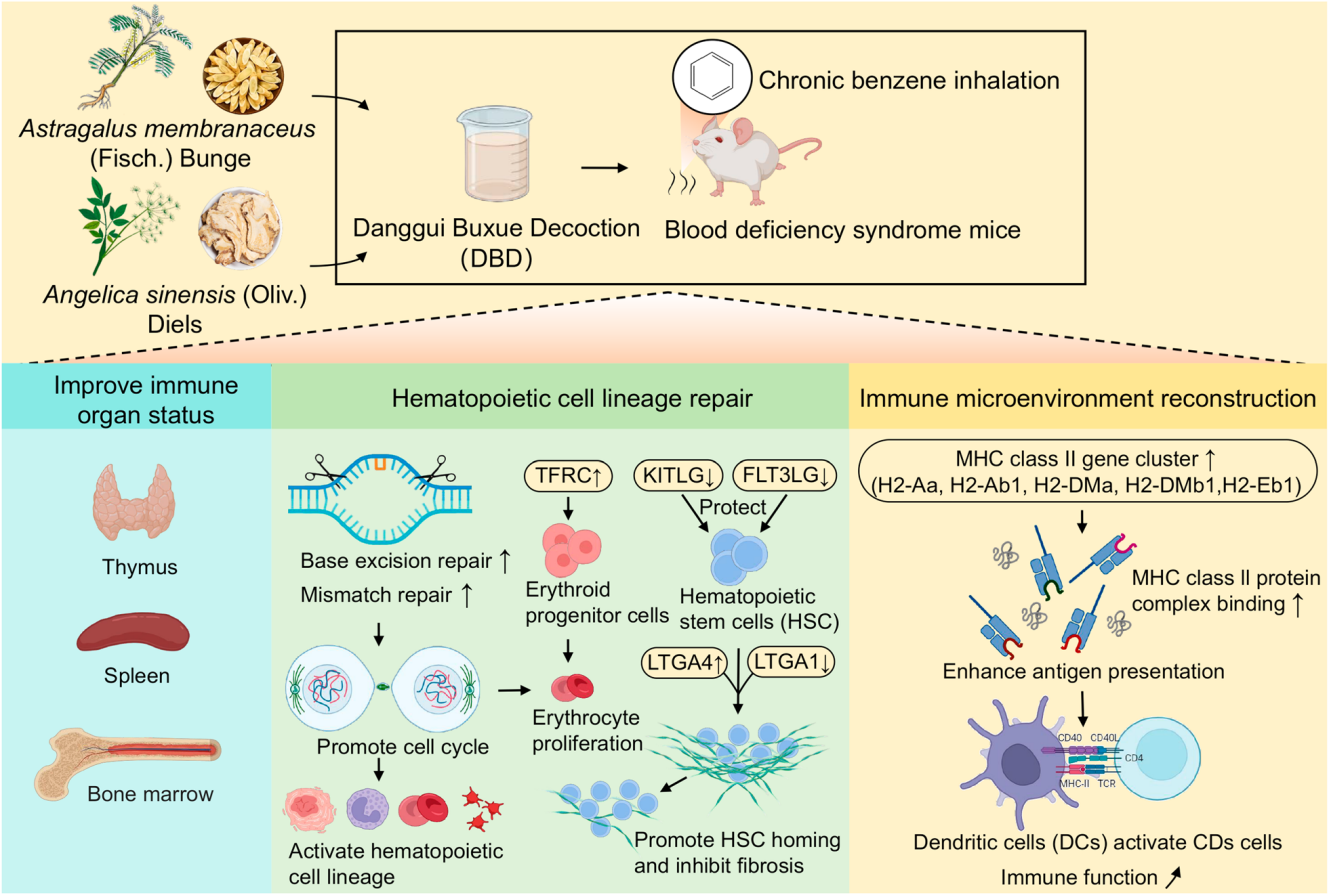

**Supplementary Information:**

The online version contains supplementary material available at 10.1186/s13020-026-01365-5.

## Introduction

Blood deficiency syndrome (BDS) is a fundamental syndrome in traditional Chinese medicine (TCM) characterized by symptoms such as pale complexion, dizziness, palpitations, blurred vision, and irregular menstruation, which are attributed primarily to insufficient blood production or chronic blood loss [1]. In modern medical contexts, the pathophysiological manifestations of BDS often align with hematopoietic disorders and immune imbalances [2], which are seen in conditions such as iron deficiency anemia, aplastic anemia, bone marrow suppression following chemotherapy or benzene exposure, and chronic inflammatory diseases [3, 4]. The core pathophysiology involves diminished hematopoietic function, aberrant cytokine signaling, and oxidative stress, leading to tissue hypoxia and multiorgan dysfunction. Although traditional treatment methods, such as iron supplements or recombinant erythropoietin (rhEPO) [5, 6], are available, limitations still exist. Androgen derivatives such as stanozolol can cause hepatotoxicity [7, 8] and masculinization [9]; moreover, rhEPO increases the risk of thrombosis and promotes tumor angiogenesis [10], and immunosuppressants can lead to osteoporosis and metabolic disorders [11]. These shortcomings highlight the need for safer alternatives. Because of its multicomponent, multitarget, synergistic therapeutic effect and low toxicity, TCM has great potential in the treatment of BDS, which has stimulated interest in the treatment of BDS with TCM [12].

**Fig. 1:**
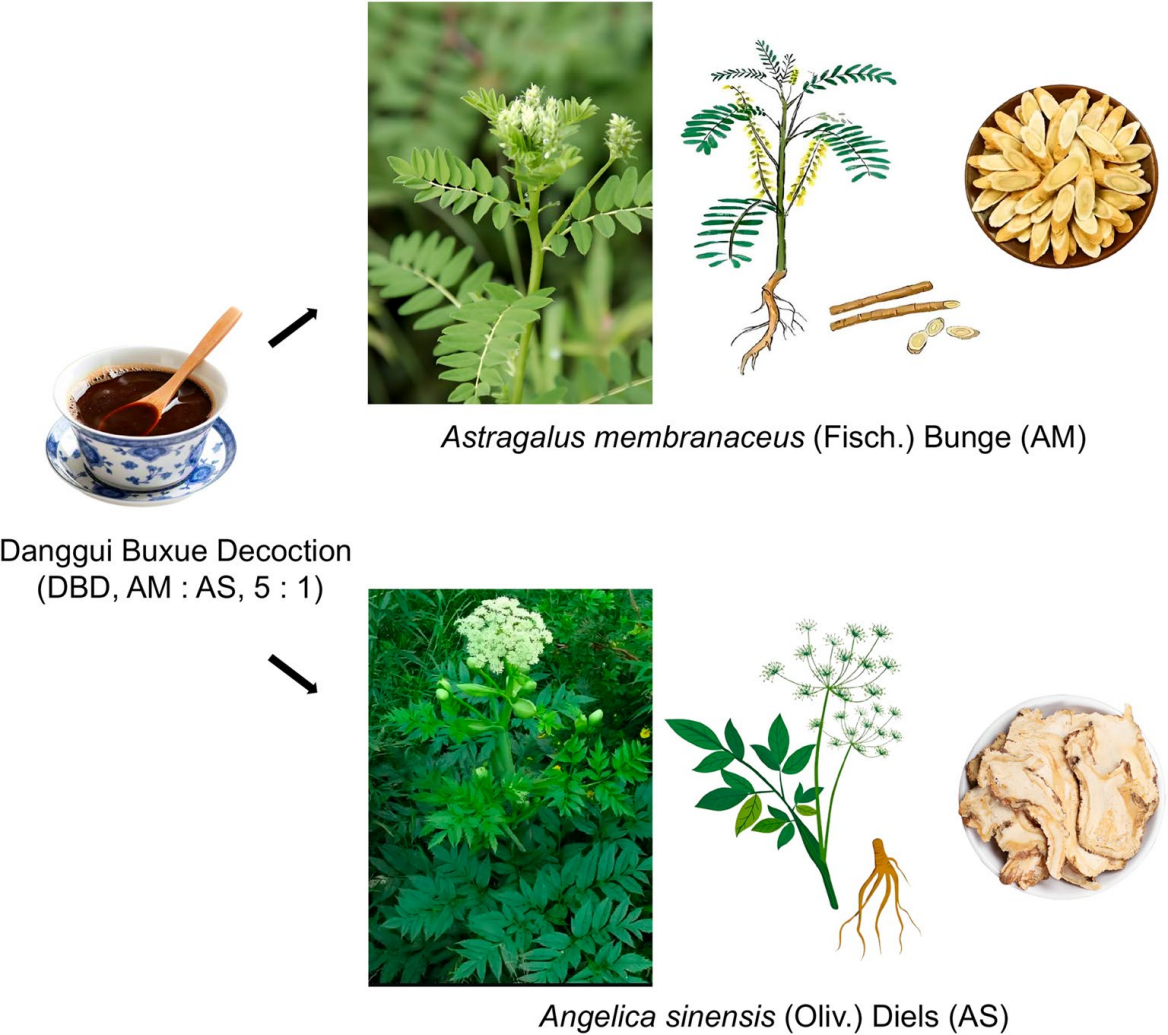
Formulation and ratio of DBD. Astragali Radix (*Astragalus membranaceus* (Fisch.) Bunge) and Angelicae Sinensis Radix (*Angelica sinensis* (Oliv.) Diels) at a 5:1 mass ratio of dried medicinal materials

Danggui Buxue Decoction (DBD) is a TCM formula commonly used to treat BDS [13, 14]. It was first documented in Li Dongyuan's “Treatise on Discrimination of Endogenous and Exogenous Diseases” (1247 CE) in the Jin and Yuan Dynasties. It is composed of Astragali Radix (*Astragalus membranaceus* (Fisch.) Bunge) and Angelicae Sinensis Radix (*Angelica sinensis* (Oliv.) Diels) at a ratio of 5:1 in terms of the weight of the medicinal materials (Chinese Pharmacopoeia 2025 Edition) (Fig. 1). DBD has significant clinical efficacy on BDS [15]. Its main bioactive ingredients include calycosin, formononetin, astragaloside IV, astragalus polysaccharides, ferulic acid, ligustilide and angelica polysaccharides [16, 17]. Clinical application has confirmed that DBD has the advantages of multiple effects and systemic regulation in the treatment of BDS. However, the efficacy of DBD has not been systematically evaluated, and there is a lack of systematic interpretation of the molecular mechanism underlying its efficacy. In particular, modern biological interpretation of the efficacy of target recognition of DBD and the regulatory target-mediated “blood tonifying immune” synergistic effect are lacking, which leads to the clinical application of DBD being limited to empirical use by doctors. This not only hinders the objective establishment of efficacy evaluation criteria but also hinders the precise use and promotion of DBD in the modern medical system. These problems urgently need to be solved by DBD research.

Proteomic analyses can monitor and respond to the actual state and functional activity of all proteins expressed by organisms in a specific state, reflecting the real physiological function, metabolic pathway, signal transduction state and response to environmental stimuli or diseases. Transcriptomics involves monitoring the transcriptional activity of genes, determining their relative expression levels, and revealing the dynamic changes in gene regulation and potential biological status by analyzing the total RNA expressed in a specific cell or tissue at a specific time. Proteomics and transcriptomics complement and verify each other and allow comprehensive and in-depth analysis of biological processes at different levels, making them favorable tools for studying the mechanism of drug-induced functional recovery in biological organisms [18, 19].

In this study, a mouse model of BDS was established through chronic benzene inhalation to recapitulate the progressive hematopoietic and immune dysfunction observed in clinical BDS. The therapeutic effects of DBD were systematically evaluated on the basis of peripheral blood parameters, histopathological changes in the thymus and spleen, bone marrow microstructure, and hematopoietic and inflammatory factor levels in vivo. Integrated proteomic and transcriptomic analyses were further employed to identify DBD-modulated targets and pathways, as well as the restoration of associated biological functions. The results demonstrated that DBD significantly improved hematopoietic and immune functions in BDS mice, effectively reversed abnormalities in peripheral blood and the bone marrow microenvironment, and regulated key hematopoietic and inflammatory factors. Mechanistically, DBD exerted systemic therapeutic benefits through synergistic actions involving hematopoietic lineage repair and immune microenvironment remodeling. These findings provide a systematic molecular basis for the clinical application of DBD and offer new insights into targeted therapeutic strategies for BDS-related disorders.

## Materials and methods

### Ethics statement

The male CD1 mice used in this study were purchased from Liaoning Changsheng Biotechnology Co., Ltd. (License No.: SCXK 2020–0001). All animal experimental protocols were approved by the institutional Experimental Animal Welfare Ethics Committee of the Heilongjiang University of Chinese Medicine, and all experiments were carried out in accordance with the Declaration of Helsinki (Approval Number: 2023062511). The mice were housed under specific pathogen-free (SPF) conditions with free access to sterile food (irradiated) and sterile water under a 12-h light/dark cycle.

### Instruments

A Vanquish™ Neo UHPLC liquid chromatograph (Thermo Fisher, USA), an EASY-nLC 1200 liquid chromatograph (Thermo Scientific, USA), an Orbitrap Astral mass spectrometer (Thermo Fisher, USA), a Tims TOF™ pro mass spectrometer (Bruker, USA), TRIzol reagent (Invitrogen, CA, USA), a NanoDrop 2000 spectrophotometer (Thermo Scientific, USA), a thermostatic drying oven (BPG-9056A, China), an SDS polyacrylamide gel electrophoresis apparatus (DYY-6C, Six One Instrument Factory Beijing City, China), an automatic digital gel image analysis system (Shanghai Tianneng Technology Co., Ltd, China), an electronic analytical balance (XSE05, Mettler Toledo, Switzerland), a table centrifuge (Sorvall ST 16R, Thermo Scientific, USA), and an ultralow temperature refrigerator (995, Thermo Scientific, USA) were used.

### Sample preparation of DBD

The prescribed doses of Astragali radix (41.30 g) and wine-steamed Angelicae sinensis radix (8.26 g) were weighed and placed in an electric hot pot. Distilled water (600 mL) was added, and the samples were soaked for 40 min. The mixture was boiled at a power of 500 W and then decocted for 80 min at a power of 120 W. After cooling to room temperature, the extract was filtered through a 160-mesh sieve. The filtrate was freeze-dried, and the resulting powder was collected.

### Preparation of the BDS mouse model

After adapting to a 12-h light/dark cycle, a temperature range of 22–25 °C, and a humidity range of 50–60% for one week, 108 healthy CD1 mice were selected for the experiments. Ninety mice were randomly selected and exposed to a 500-ppm benzene environment for 8 h per day for 4 weeks to establish a BDS model. The remaining 18 healthy mice were used as the control group, with the same feeding conditions except that they were not exposed to benzene.

### Treatment of DBS mice

After the BDS model was successfully established, the 90 model mice were randomly and equally divided into the following groups: the model group (Group M), low-dose DBD group (DBD-L, 200 mg/kg), medium-dose DBD group (DBD-M, 400 mg/kg), high-dose DBD group (DBD-H, 800 mg/kg), and stanozolol treatment group (STZ, 0.78 mg/kg). Each group contained 18 mice (6 for efficacy evaluation, 6 for proteomics, and 6 for transcriptomics). All groups received daily oral gavage at 9:00 AM for 4 weeks according to their respective treatment protocols, and control group mice were given equivalent volumes of distilled water.

### Biological sample collection and processing

After treatment, 200 μL of blood was collected from each mouse into EDTA-coated tubes and gently mixed to prevent coagulation for complete blood count analysis. The remaining blood was placed in standard serum collection tubes for serum preparation and subsequent ELISA kit detection. The thymus and spleen were excised for pathological examination, organ index determination (thymus index = thymus wet weight (mg)/body weight (g) × 100; spleen index = spleen wet weight (mg)/body weight (g) × 100), and multi-omics studies.

### DIA proteomics analysis

Mouse spleens were collected and fully ground in liquid nitrogen for protein extraction. Sample quality control was performed using SDS–polyacrylamide gel electrophoresis (SDS–PAGE). After confirming the quality of the protein extracts, the proteins were enzymatically digested into peptides and desalted. Prepared samples were subjected to data-independent acquisition (DIA) proteomic analysis using a Vanquish neoantigen chromatography system coupled with an Orbitrap Astral mass spectrometer. For LC–MS/MS identification, DIA technology was used to collect mass spectrometry data for each sample. Specific parameter settings are provided in the Supplementary Materials (Supplementary Table 1). The acquired DIA raw data were imported into Spectronaut Pulsar 18.4 software (Biognosys) for analysis and protein identification. Peak matching, quantitative information extraction, and statistical analysis were performed. Differential expression of protein in mouse spleens between groups was evaluated using the fold change (FC) combined with p values obtained from t tests. Proteins meeting the criteria of a p value < 0.05 and an FC ≥ 2.0 or ≤ 0.5 were identified as differentially expressed proteins. These proteins were subjected to Gene Ontology (GO) enrichment analysis and Kyoto Encyclopedia of Genes and Genomes (KEGG) pathway analysis to determine key pathways and targets of DBD in the treatment of BDS.

### RNA extraction and library construction

Total RNA was extracted using the TRIzol reagent according to the manufacturer's instructions. RNA purity and concentration were determined using a NanoDrop 2000 spectrophotometer (Thermo Scientific, USA), and RNA integrity was assessed with an Agilent 2100 Bioanalyzer (Agilent Technologies, Santa Clara, CA, USA). Transcriptome libraries were constructed using the VAHTS Universal V10 RNA-seq Library Prep Kit (Premixed Version) following the manufacturer's protocol. Transcriptome sequencing and analysis were performed by Shanghai OE Biotech Co., Ltd. (Shanghai, China).

### Transcriptome sequencing analysis methods

RNA sequencing and differential gene expression analysis were conducted on the Illumina NovaSeq 6000 platform. The raw data were processed using fastp software to remove low-quality reads, with the clean reads used for subsequent analysis. HISAT2 software was used to align the reads to the reference genome and calculate gene expression levels (FPKM). Read counts per gene were obtained using HTSeq-count. Principal component analysis (PCA) of gene counts was performed in R (v 3.2.0) to evaluate biological replicates. Differential expression analysis was carried out with DESeq2 software, defining genes meeting the thresholds of a q-value < 0.05 and |fold change|> 2 (equivalent to |log2FC|> 1) as differentially expressed genes (DEGs).

### Statistical analyses

Statistical analyses were performed using GraphPad Prism 8.0 software (San Diego, CA, USA). Data are expressed as the mean ± SD and were analyzed by one-way ANOVA and unpaired two-tailed t tests for comparisons between groups. Differences were considered to be statistically significant when the p value was < 0.05.

## Results

### DBD significantly ameliorates hematopoietic dysfunction and restores peripheral blood homeostasis in BDS mice

**Fig. 2:**
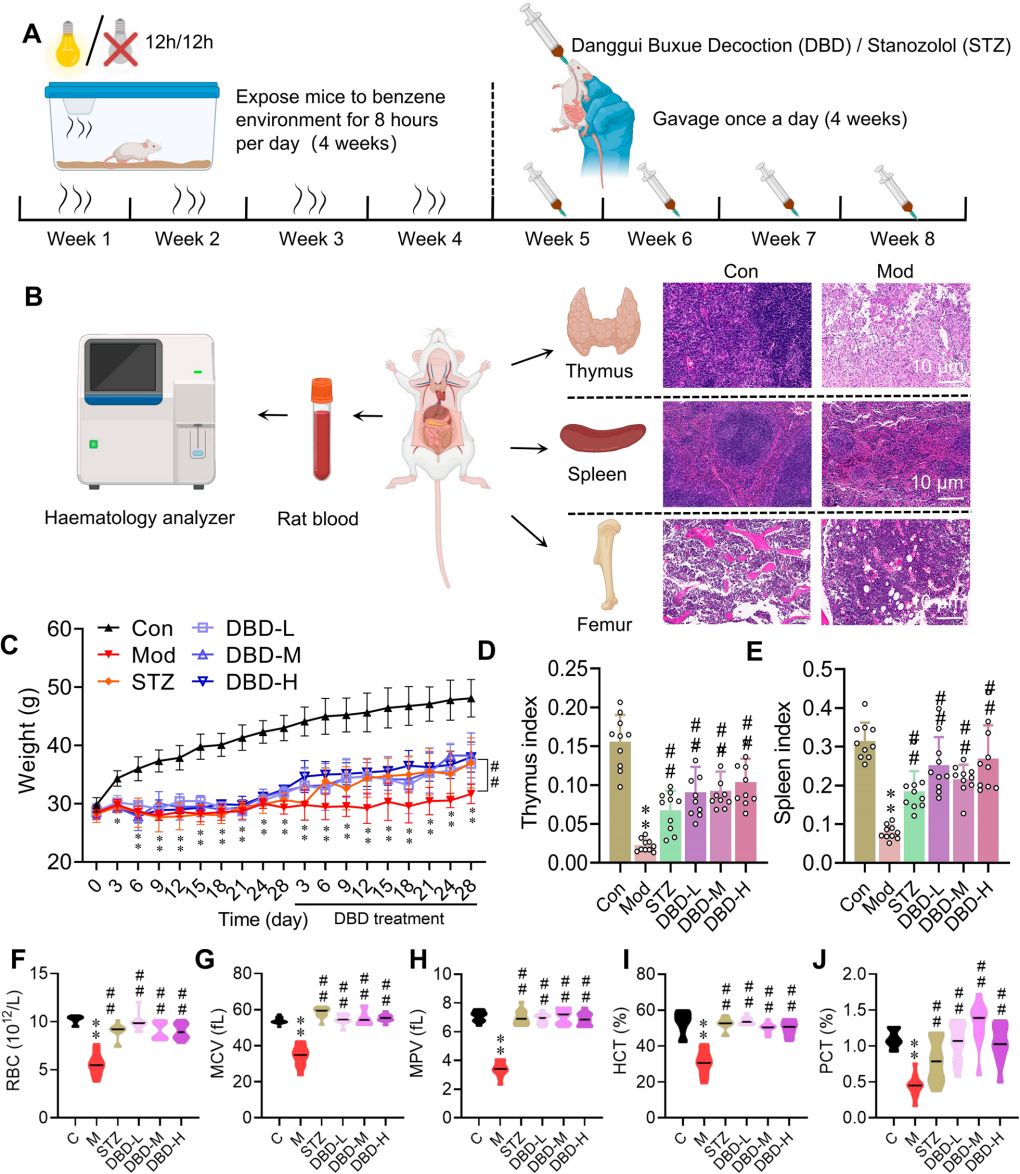
DBD significantly restored body weight and immune organ indices in BDS mice and increased the number and proportion of blood cells per unit volume of blood. **A** Flowchart of the development of the BDS mouse model and treatment protocol. **B** Sample collection and evaluation of the BDS mouse model. The pathological states of immune organs (thymus and spleen) and the bone marrow microenvironment significantly differed. **C** DBD significantly restored body weight in mice (**D**, **E**) and the indices of immune organs (thymus and spleen). **F**–**J** DBD significantly increased the number of red blood cells (RBCs) per unit volume of blood in BDS mice and elevated the mean corpuscular volume (MCV), mean platelet volume (MPV), hematocrit (HCT), and plateletcrit (PCT)

The experimental timeline (Fig. 2A) involved exposing healthy CD1 mice to benzene (8 h/day, 4 weeks) to establish a chronic benzene inhalation-induced blood deficiency model. After successful modeling, daily oral gavage of DBD was administered for 4 weeks. Body weight was monitored throughout the study, with collection of blood, thymus, spleen, and femur for analysis on the final day (Fig. 2B). During modeling, compared with control mice, benzene-exposed mice had significantly lower body weights (p < 0.05). DBD treatment progressively reversed this weight loss, yielding statistically significant differences between the treatment and model groups (Fig. 2C, p < 0.05). DBD significantly increased the thymic and splenic indices (Fig. 2D, E). Peripheral blood analysis revealed that compared with control mice, DBD-treated mice presented elevated red blood cell (RBC) counts. Key indices—mean corpuscular volume (MCV), mean platelet volume (MPV), hematocrit (HCT), and plateletcrit (PCT)—were significantly restored, approaching normal physiological ranges (Fig. 2F–J). These results collectively demonstrate the efficacy of DBD in ameliorating clinical signs and normalizing hematological profiles in blood-deficient mice.

### DBD promotes the systemic restoration of hematopoietic and immune organs

**Fig. 3:**
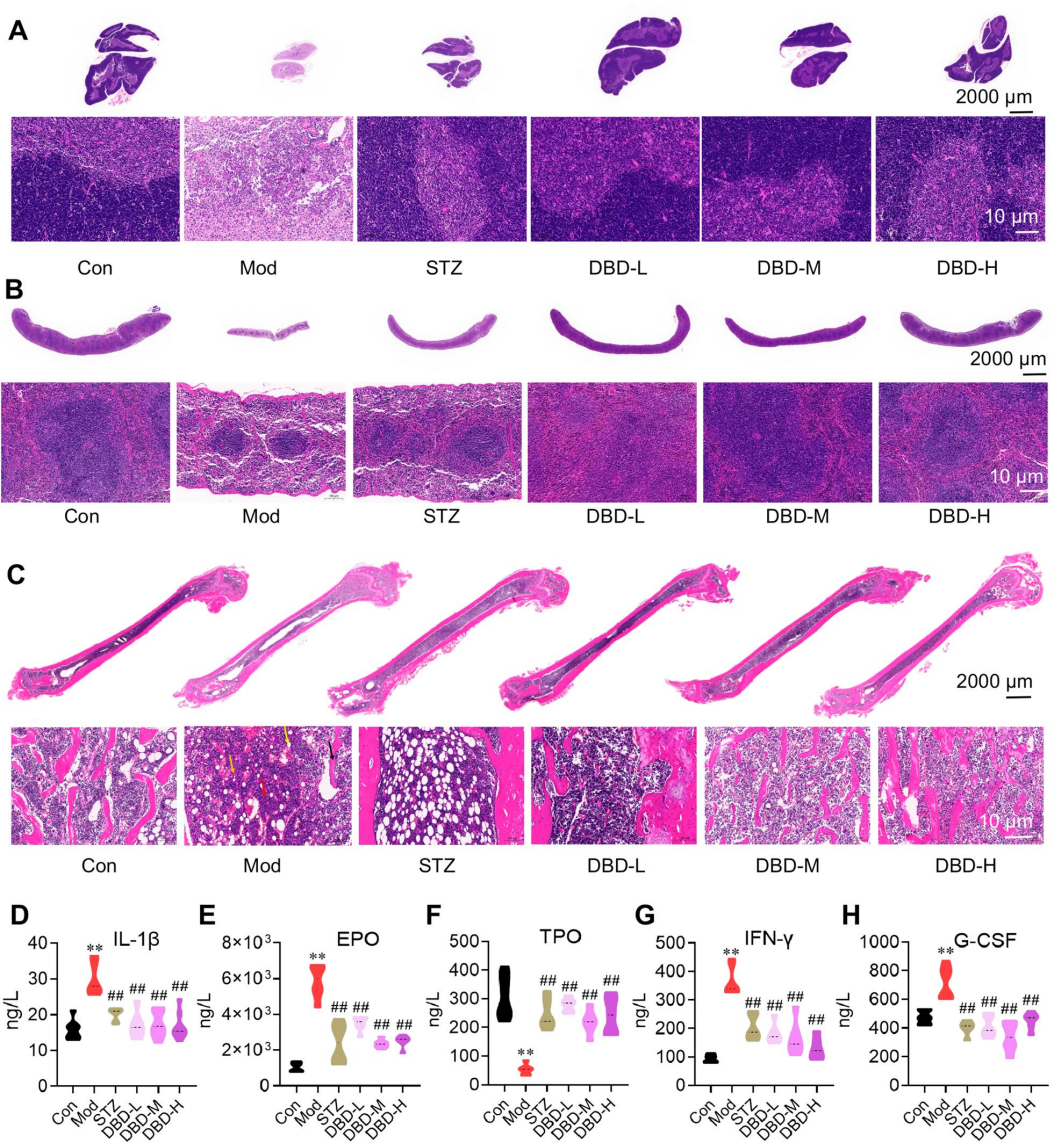
DBD significantly improved the pathological states of the thymus and spleen, restored the bone marrow microenvironment in femurs, and enhanced immune responses in BDS mice (all groups, n = 6; Con: control group; Mod: model group; STZ: stanozolol group; DBD-L: low-dose DBD group; DBD-M: medium-dose DBD group; DBD-H: high-dose DBD group; compared with the Con group, * *p* < 0.05, ** *p* < 0.01; compared with the Mod group, # *p* < 0.05, ##* p* < 0.01, means ± SDs). **A** DBD significantly ameliorated the atrophic state of the thymus in BDS mice, improved the thymic tissue cellular environment, and restored immune function. **B** DBD significantly improved the atrophic state of the spleen in BDS mice, ameliorated the cellular environment of the splenic tissue, and restored hematopoietic and immune functions. **C** DBD significantly improved the bone marrow microenvironment in the femurs of BDS mice, reduced inflammatory infiltration in the bone marrow, and enhanced hematopoietic function. **D**–**H** DBD significantly restored the levels of interleukin-1β (IL-1β), erythropoietin (EPO), thrombopoietin (TPO), interferon-γ (IFN-γ), and granulocyte colony-stimulating factor (G-CSF) in the blood of BDS mice

Following treatment completion, pathological examinations were performed on thymus, spleen, and femur tissues. Compared with control mice, model mice exhibited substantial thymic atrophy characterized by markedly reduced lymphocytes (both small and large types) in cortical and medullary regions, sparse cellular arrangement, blurred corticomedullary boundaries, increased medullary vasculature, and significant interstitial fibrotic hyperplasia. DBD treatment at all doses effectively reversed these pathological changes (Fig. 3A).

Similarly, the spleen, which serves hematopoietic and blood reservoir functions, showed significant atrophy in model mice. The white pulp displayed fewer shrunken splenic nodules, whereas the red pulp exhibited localized hyperplasia featuring increased granulocytes, few macrophages, and elevated megakaryocyte counts. DBD significantly improved the spleen status mentioned above. The white and red pulp of the spleen had a clear boundary and was evenly distributed in the spleen tissue, with good morphology. Red pulp macrophages were abundant, and the splenic nodules were in a normal state (Fig. 3B).

Bone marrow, the essential hematopoietic “production factory” for blood cells, demonstrated pathological alterations in model mice: increased granulocytes/megakaryocytes, reduced hematopoietic cells at various developmental stages (replaced by adipocytes), and diminished fragmented bone spicules. DBD treatment restored the marrow microenvironment, with uniformly distributed spicules, reticular cell/fiber networks supporting diverse developing blood cells, minimal megakaryocytes/adipocytes/granulocytes, and abundant sinusoids resembling those in control conditions (Fig. 3C).

In addition, after four weeks of DBD treatment of BDS mice, serum levels of hematopoietic growth factors, inflammatory cytokines, and immunoregulatory factors were measured across groups. The results demonstrated that DBD significantly increased the blood concentration of thrombopoietin (TPO) but markedly decreased the levels of interleukin-1β (IL-1β), erythropoietin (EPO), granulocyte colony-stimulating factor (G-CSF), and interferon-γ (IFN-γ) (Fig. 3D–H).

These changes indicate the dual therapeutic efficacy of DBD: it reverses benzene-induced “hematopoietic suppression” and “inflammatory hyperactivation” by enhancing hematopoietic parenchymal function and reconstructing the immune microenvironment, thereby achieving concurrent blood-replenishing and immunoenhancing effects.

### Proteomic profiling reveals DBD-mediated restoration of hematopoietic lineage and antigen presentation in BDS mice

**Fig. 4:**
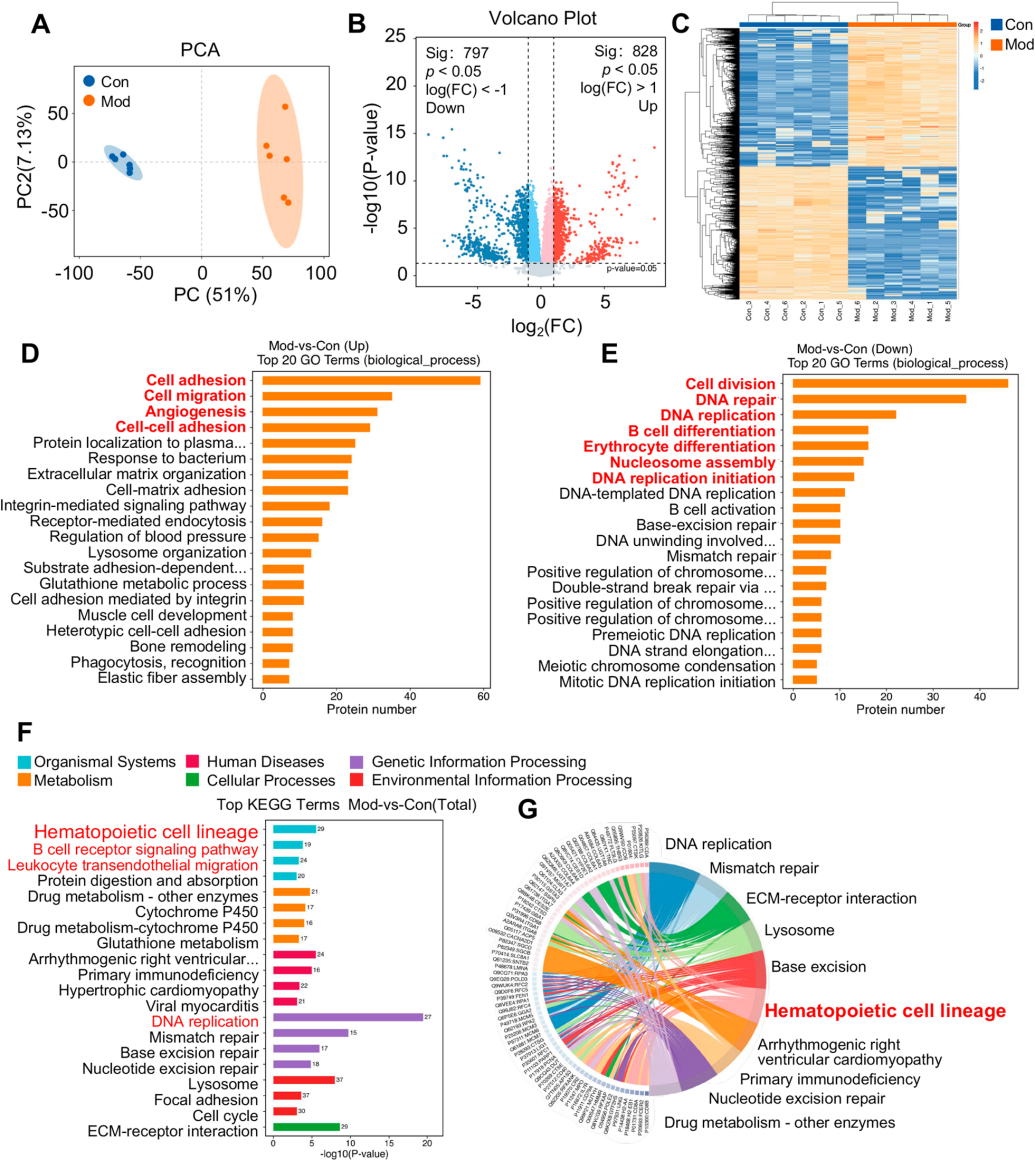
Screening and analysis of differentially expressed proteins in the spleens of blood deficiency model mice. **A** PCA of spleen protein expression in the Con group and Mod group. **B** Differential protein analysis of the spleen between the Con group and Mod group. **C** Cluster heatmap analysis of differentially expressed proteins. GO enrichment analysis of abnormally high/low expression proteins in the **D**, **E** Mod group. **F** KEGG enrichment analysis of differentially expressed proteins. **G** The top 10 pathways with significant abnormalities in the Mod group mice (ranked with the lowest p value)

Proteomic analysis of the spleens of the BDS mice revealed significant changes in protein expression profiles compared with those of the control group (Fig. 4A). This included 828 proteins whose expression was abnormally elevated and 797 proteins whose expression was decreased. The results of the differential expression analysis are shown in Fig. 4B, C. These findings suggest that certain biological functions in BDS mice induced by benzene inhalation undergo significant changes. GO analysis revealed that core hematopoietic processes, including cell division, DNA replication/repair, and erythroid/B-cell differentiation, were significantly downregulated, reflecting direct benzene toxicity causing DNA damage, cell cycle arrest, and impaired differentiation. Conversely, compensatory processes involving cell adhesion/migration, angiogenesis, extracellular matrix reorganization, antibacterial/phagocytic responses, and glutathione metabolism were upregulated. These findings indicate that spleen-specific stress adaptation to bone marrow failure features microenvironment remodeling, inflammation, and oxidative defense (Fig. 4D, E). KEGG pathway analysis confirmed these findings, highlighting the suppression of the hematopoietic cell lineage pathway and enrichment of adhesion, glutathione, and immune–inflammatory pathways (Fig. 4F–G). This comprehensive profile elucidates the molecular basis of benzene-induced hematopoietic failure and provides a foundation for understanding therapeutic mechanisms.

**Fig. 5:**
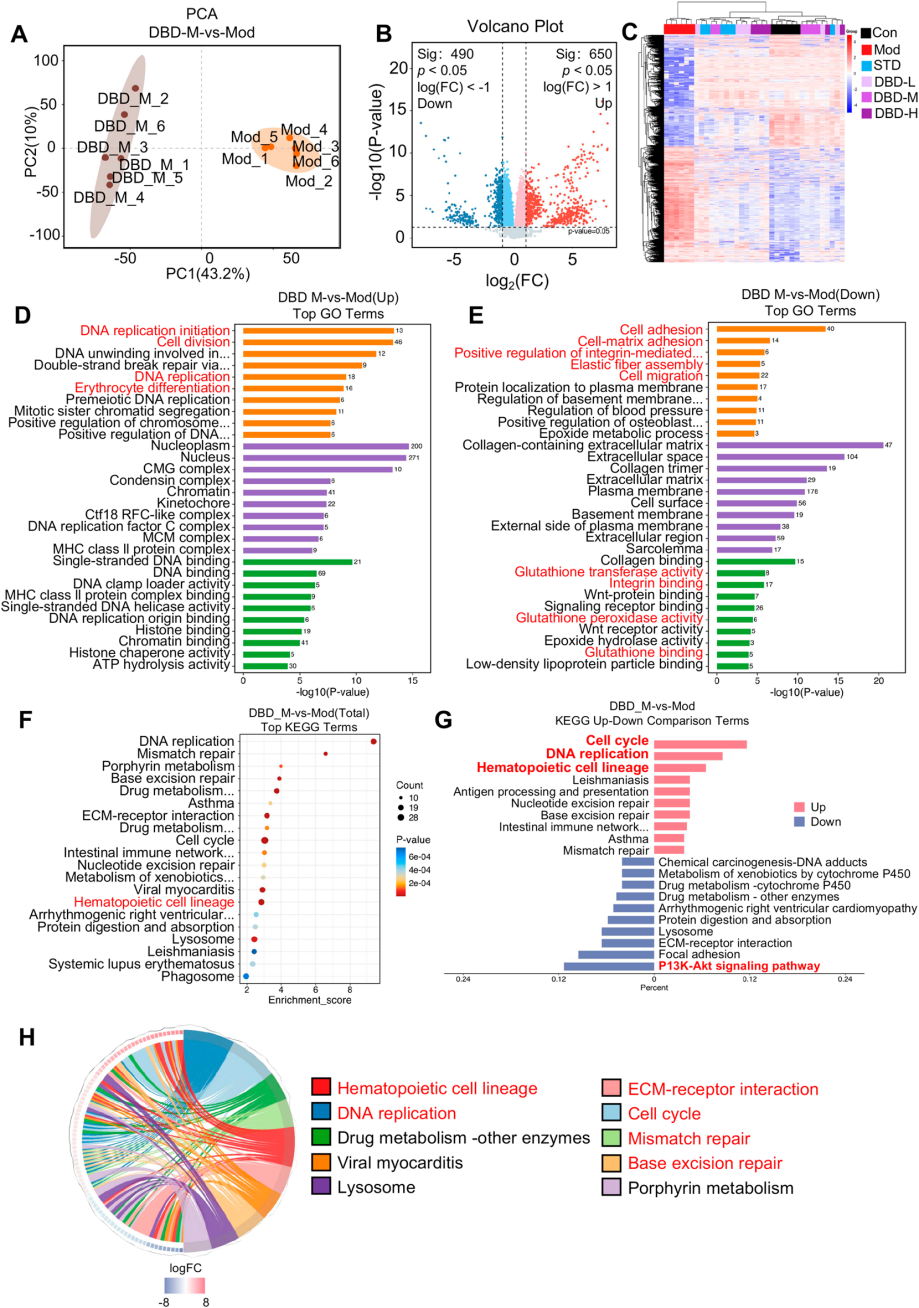
Proteomic analysis revealed the effect of DBD on spleen protein expression in BDS mice. **A** PCA of spleen protein expression in the DBD-M group and Mod group. **B** Differential expression analysis of spleen proteins between the DBD-M group and the Mod group. **C** Heatmap analysis of differential protein expression in each group. **D** GO enrichment analysis of proteins significantly upregulated by DBD. **E** GO enrichment analysis of proteins significantly downregulated by DBD. **F** KEGG enrichment analysis of differentially expressed proteins. **G** Enrichment analysis of pathways upregulated and downregulated by DBD. **H** The top 10 pathways significantly regulated by DBD (with the lowest p value)

To elucidate the therapeutic mechanism of DBD on chronic benzene-induced BDS, on the basis of the results of the efficacy evaluation of DBD, we selected the DBD-M group for proteomic study and compared the proteomic characteristics of the spleens of the DBD-M group and Mod mice. PCA revealed significant differences in the spleen protein expression profiles between the two groups of mice (PC1 = 43.2%, PC2 = 10.0%). Under the regulation of DBD, 650 proteins were significantly upregulated, and 490 proteins were significantly downregulated (*p* < 0.05, FC > 2). An expression heatmap analysis of these differentially expressed proteins in each group is shown in Fig. 5C. GO enrichment analysis demonstrated the precise targeting of pathological features by DBD. DBD prominently repaired benzene-induced DNA damage and reactivated stalled cell cycles, as evidenced by upregulated DNA replication functions (initiation, general replication, unwinding, and premeiotic replication) and enhanced double-strand break repair and DNA repair regulation. This restored proliferative capacity by increasing cell division and sister chromatid segregation, supported by improved chromosomal stability (chromosome regulation, condensin complex, and kinetochore) and fully reassembled replication machinery (CMG/MCM/DNA replication Factor C complexes, clamp loading, origin binding, and single-stranded DNA binding/helicase activities). Chromatin regulation (histone/chromatin binding and chaperone activity) and nuclear maintenance were simultaneously reinforced, collectively reversing DNA damage accumulation, repair failure, replication arrest, and proliferation suppression, thereby reactivating splenic hematopoietic stem/progenitor cells (Fig. 5D).

Critically, DBD directly targeted the core pathology of blood deficiency, hematopoietic impairment, by significantly upregulating erythrocyte differentiation. These findings molecularly validate the blood-replenishing efficacy of DBD by specifically repairing impaired erythropoiesis. DBD also enhanced immune recognition via upregulation of MHC class II complexes and their binding capacity, suggesting improved antigen presentation by B cells/dendritic cells. Furthermore, DBD suppressed pathological hyperresponses, such as decreased cell adhesion/migration (cell–matrix adhesion and integrin-mediated signaling), ECM remodeling (elastic fiber assembly, collagen-containing ECM, and collagen/integrin binding), and oxidative stress (glutathione transferase/peroxidase activity and glutathione binding). These changes indicate that DBD alleviates compensatory extramedullary hematopoiesis, fibrotic tendencies, and oxidative damage, restoring microenvironmental homeostasis. Additional normalization included adjusted membrane signaling (Wnt-protein/receptor activities) and elevated ATP hydrolysis for energy support (Fig. 5E).

The KEGG enrichment analysis yielded the same results. DBD treatment of BDS mice upregulated DNA repair pathways, particularly base/nucleotide excision repair and mismatch repair. The cell cycle process is activated through the DNA replication pathway. It is crucial that the upregulation of the hematopoietic cell lineage by DBD confirms the restoration of blood cell proliferation and differentiation. Moreover, DBD reduces the burden of exogenous detoxification, downregulates cytochrome P450 metabolism, and inhibits ECM receptor interactions, focal adhesion, and PI3K/Akt signaling, resulting in pathological remodeling and restoring spleen morphology and function. More importantly, DBD enhances immune recognition, antigen processing, and antigen presentation simultaneously (Fig. 5F–G).

In summary, DBD repairs DNA damage, restarts proliferation, and activates erythroid differentiation through multitarget regulation while normalizing the stress response and immune dysfunction. This comprehensive molecular mechanism validates the overall therapeutic effect of DBD on benzene-induced BDS mice.

### Validation of dual mechanisms by transcriptomic analysis: DBD restores hematopoietic homeostasis and remodels the immune response through TFRC/KITLG/FLT3LG/LGTA1 and MHC class II gene clusters

**Fig. 6:**
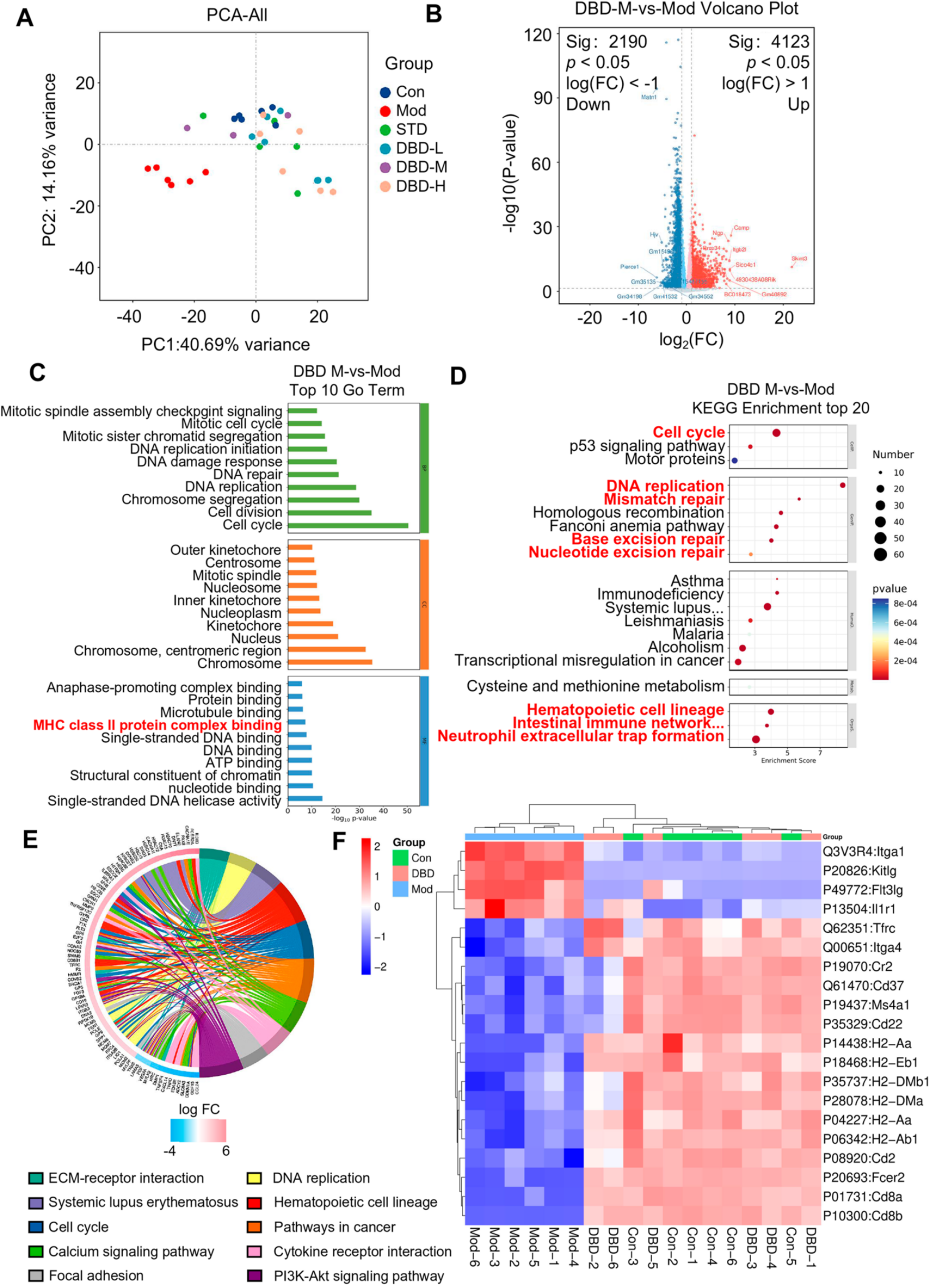
Transcriptomic data have revealed that the effects of DBD on DBS are achieved primarily through the promotion of hematopoietic cell lineage repair and immune microenvironment remodeling. **A** PCA of spleen gene expression in all groups of mice. **B** Differential gene expression analysis between the DBD treatment group and the Mod group. **C** GO enrichment analysis of DEGs regulated by DBD. **D** KEGG enrichment analysis of genes regulated by DBD. **E** Functional enrichment analysis of the top 10 pathways (p values) associated with the DBD-regulated genes. **F** Thermal map analysis of gene expression regulated by DBD in hematopoietic cell lineage repair and immune microenvironment remodeling

To further validate and explore the regulatory effect of DBD on gene expression in DBS mice, we conducted a transcriptomic analysis of the spleens of all groups of mice. After treatment, the gene expression profiles of the spleens of the Mod group and Con group mice were still significantly different, and the gene expression profiles of the different DBD treatment groups were similar to those of the Con group (Fig. 6A). Among them, DBD significantly upregulated 4123 genes and downregulated 2190 genes (Fig. 6B). GO and KEGG enrichment analyses were performed on these genes, and the results revealed that DBD can synergistically restore hematopoietic immune homeostasis in the spleen of chronic benzene-induced blood deficiency mice through multitarget gene regulation (Fig. 6C–E).

GO enrichment analysis demonstrated that DBD markedly upregulated the expression of gene clusters involved in antigen-binding α/β chains and MHC class II antigen presentation (including H2-Aa, H2-Ab1, H2-DMa, H2-DMb1, and H2-Eb1), collectively enhancing the functionality of antigen-presenting cells. In parallel, DBD activates both B and T-cell networks, leading to remodeling of the immune microenvironment. Mechanistically, DBD upregulates Cd22 and Cd37 expression to modulate B-cell signaling, increases Cr2-mediated complement internalization, upregulates Cd8a and Cd8b expression to amplify cytotoxic T-cell responses, increases FCER2 expression to regulate B-cell differentiation, and promotes the activation of Ms4a1-positive B cells.

KEGG enrichment analysis further revealed that DBD finely tunes stem cell homeostasis by downregulating Kitlg (SCF) and FLT3LG, thereby preventing hematopoietic stem cell exhaustion from overdifferentiation while upregulating ITGA4 (integrin α4) to facilitate VCAM-1⁺ homing and downregulating Itga1 to inhibit collagen binding and fibrotic progression. These observations align with proteomic profiling, confirming that DBD mitigates compensatory extramedullary hematopoiesis, a predisposition to fibrosis, and oxidative stress, ultimately restoring microenvironmental homeostasis and increasing immune recognition, antigen processing, and antigen presentation.

**Fig. 7:**
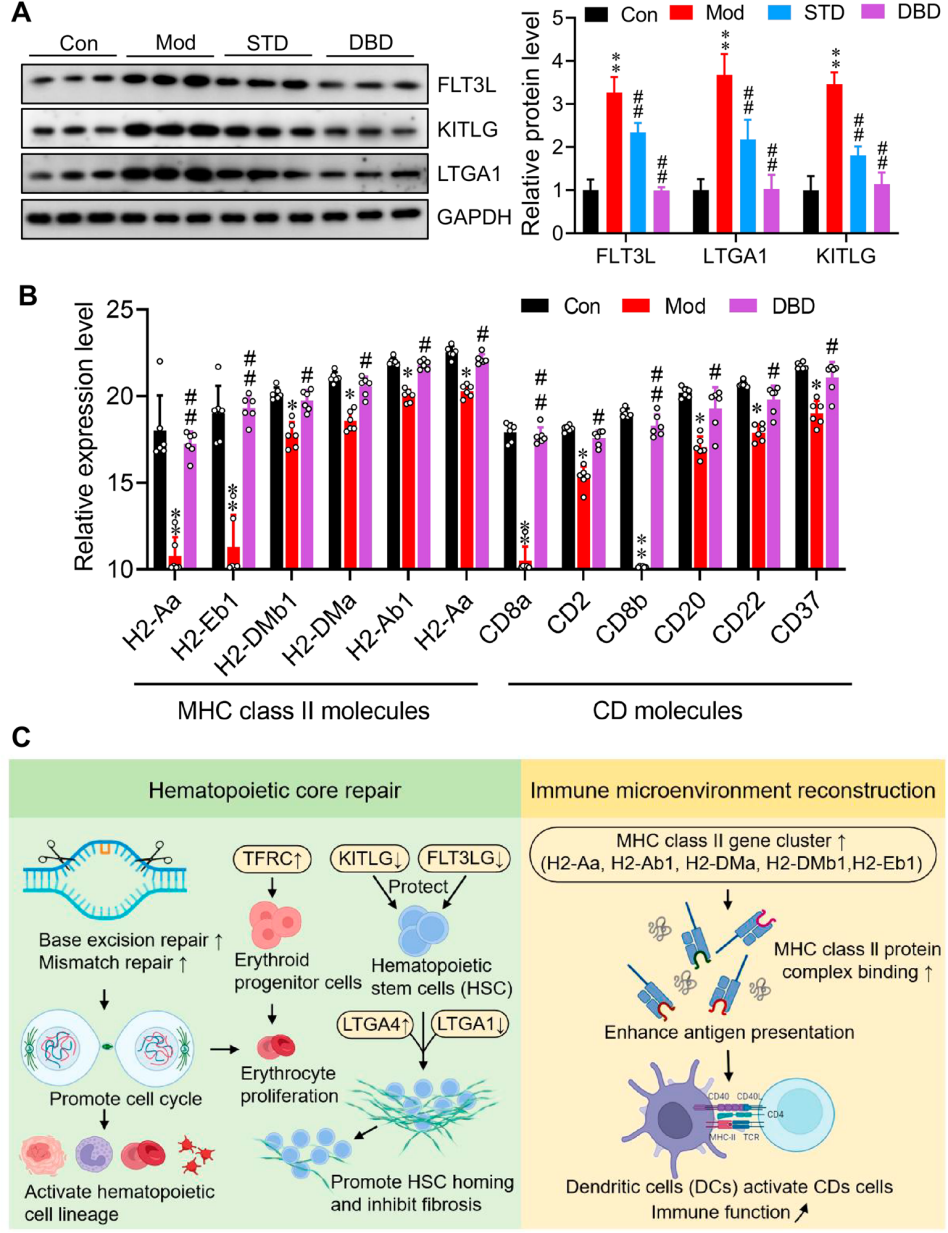
DBD exerts therapeutic effects on DBS by repairing hematopoietic cell lineages and reshaping the immune microenvironment. **A** DBD significantly downregulates the protein expression of FLT3L, KITLG, and LTGA1. **B** DBD significantly upregulates the expression of MHC class II gene family members and increases the expression level of MHC class II molecules. In addition, it promotes the activation of immune T/B cells to generate CD molecules. **C** DBD can significantly restore immune organs and bone marrow suppression, mainly by repairing hematopoietic cell lineages and reshaping the immune microenvironment to exert therapeutic effects on DBS

In summary, transcriptomic profiling revealed that DBD exerts its therapeutic effects on DBS primarily through two interconnected mechanisms: hematopoietic lineage restoration and immune microenvironment remodeling. First, regarding hematopoietic lineage repair, DBD upregulates TFRC to promote erythroid progenitor expansion and iron-dependent erythropoiesis while downregulating Kitlg and Flt3lg to prevent excessive depletion of hematopoietic stem cells, thereby maintaining the sustainability of the stem cell pool. Concurrently, the upregulation of ITGA4 and the downregulation of ITGA1 collectively facilitate stem cell homing, suppress fibrosis progression, and preserve physiological hematopoietic niches. Western blot experiments confirmed the results of our analysis (Fig. 7A). Second, in terms of immune microenvironment remodeling, DBD enhances the expression of MHC class II gene family members, which strengthens MHC II-mediated antigen presentation and promotes T/B-cell activation (Fig. 7B). This results in comprehensive remodeling of the immune microenvironment and increased secretion of hematopoietic growth factors. Comprehensive remodeling of the immune microenvironment is promoted, and immune function is restored (Fig. 7C).

## Discussion

As a traditional Chinese medicine formula, DBD has the advantages of multiple targets, low toxicity, and systemic regulation. It has a significant therapeutic effect on BDS and can effectively address the limitations of single efficacy and notable adverse effects associated with conventional drugs used in clinical treatment of BDS. A comprehensive evaluation of the efficacy of DBD in treating BDS and elucidation of its therapeutic mechanisms will greatly promote its clinical application and dissemination while also providing a foundation for the future discovery of bioactive compounds derived from DBD.

In this study, we established a chronic benzene inhalation-induced BDS mouse model to systematically evaluate the holistic efficacy of DBD in improving peripheral blood profiles, repairing hematopoietic organ damage, restoring the bone marrow microenvironment, increasing hematopoietic factor levels, and suppressing inflammation. Integrated proteomics and transcriptomics were employed to elucidate the multitarget regulation of the hematopoietic cell lineage and immune homeostasis. Our findings suggest that DBD may facilitate recovery from benzene-induced DNA damage, restart the cell cycle, and specifically promote the differentiation and proliferation of hematopoietic stem cells, with the core manifestation being the restoration of hematopoietic cell lineages. Key targets regulated by DBD include upregulation of TFRC to promote iron-dependent erythropoiesis, downregulation of FLT3LG and KITLG to protect hematopoietic stem cells, and modulation of ITGA4(↑)/ITGA1(↓) to promote hematopoietic stem cell homing and inhibit fibrosis. The regulation of these targets collectively achieves precise modulation of “hematopoietic–immune recovery”. Furthermore, DBD significantly upregulates the expression of the MHC class II gene family, enhances antigen presentation processes, promotes immune cell activation, remodels the immune microenvironment, and restores immune function. Concurrently, DBD suppresses compensatory adhesion/migration, hyperactive inflammation, and oxidative stress in the spleen. These results not only advance the clinical application of DBD but also provide clinically feasible targets and pathways for BDS treatment, establishing a therapeutic paradigm extendable to other hematopoietic and immune disorders.

Erythropoiesis disorder is the most intuitive manifestation of BDS (pale face, fatigue, and dizziness) and is highly similar to anemia in Western medicine. As the core pathway of hematopoiesis, the hematopoietic cell lineage is involved in the differentiation and maturation of hematopoietic stem cells into erythrocytes, granulocytes, megakaryocytes/platelets and lymphoid cells (B/T cells) [20–22]. Benzene metabolites inhibit the key links of erythrocytic differentiation [23, 24] and B-cell differentiation [25] in this pathway by interfering with DNA replication and repair, resulting in abnormal peripheral blood [26, 27]. In this study, we revealed that DBD significantly upregulated the hematopoietic cell lineage pathway by activating DNA replication, base/nucleotide excision and repair; removing cell cycle arrest; and restoring the differentiation ability of multilineage hematopoietic cells, thus reversing the hematopoietic failure of BDS from the source.

The MHC class II protein complex is expressed by antigen-presenting cells (APCs) [28, 29], and its function depends on the coexpression of the h2-aa/h2-ab1 (encoding an antigen binding groove α/β chain) and h2-dma/h2-dmb1/h2-eb1 (encoding a molecular chaperone) gene clusters [30, 31]. DBD significantly upregulated these genes, promoted the assembly and presentation of the MHC II antigen peptide complex, and enhanced T-cell activation. This can not only improve the low immune recognition function of model mice and upregulate antigen processing and presentation pathways but also indirectly promote the recovery of the hematopoietic microenvironment through the secretion of hematopoietic factors (such as GM-CSF) by immune cells to regulate “immune hematopoietic” crosstalk [32].

The transferrin receptor (TFRC) serves as the core switch for iron uptake in erythroid progenitor cells. TFRC mediates the endocytosis of transferrin iron complexes, directly driving hemoglobin synthesis and red blood cell maturation [33]. DBD significantly upregulates TFRC, improves erythroid differentiation disorder in BDS, increases the levels of RBCs and HGB in the blood, and improves oxygen-carrying function to alleviate pale complexion and fatigue. KIT ligand (KITLG), also known as stem cell factor (SCF), is a key multifunctional cytokine that plays a central regulatory role in hematopoiesis, immunity, reproduction, and pigment formation [34, 35]. Its function is achieved by binding and activating the tyrosine kinase receptor (c-Kit) on the cell surface [36, 37]. KITLG binds to c-Kit receptors to maintain the self-renewal of hematopoietic stem cells (HSCs) [38, 39]. Benzene toxicity leads to the overactivity of c-Kit signaling. DBD downregulates TFRC to prevent excessive depletion of HSCs and ensure the sustainability of stem cell pools. FLT3LG (FLT3 ligand) promotes the proliferation and differentiation of lymphoid progenitor cells [40, 41]. DBD downregulates FLT3LG and works synergistically with KITLG to prevent HSC depletion while also preventing inflammation imbalance caused by excessive activation of the lymphatic system. Integrin alpha 4 (ITGA4) mediates the binding of HSCs to the bone marrow/spleen VCAM-1⁺ niche [42, 43]. Integrin alpha 1 (ITGA1) mediates the abnormal adhesion of HSCs to the collagen matrix, promoting fibrosis [44]. DBD specifically upregulates ITGA4 and downregulates ITGA1 to promote HSC homing, accelerate hematopoietic function repair, and inhibit spleen and bone marrow fibrosis and collagen deposition, protecting the normal hematopoietic space.

This study has certain limitations that should be acknowledged. First, the BDS model induced by chronic benzene inhalation primarily reflects “toxin-induced blood impairment”, which is clinically relevant to conditions such as benzene poisoning or chemotherapy-induced myelosuppression. However, it may not fully capture the etiological complexity of BDS in traditional Chinese medicine, which can arise from chronic blood loss, spleen-stomach weakness, or qi-blood deficiency. Thus, the translational relevance of our findings should be interpreted within the context of toxin-mediated hematopoietic injury. Second, while our integrated omics approach provides a comprehensive mapping of potential mechanisms, the conclusions drawn remain largely hypothetical and require further functional validation. In future studies, we plan to perform colony-forming unit assays, iron metabolism tests, flow cytometry, and construct genetic or pharmacological perturbation models to further elucidate the functional roles and interactions of key targets such as TFRC, KITLG, FLT3LG, ITGA4, and MHC II components.

Despite these limitations, this study is the first to systematically elucidate the dual regulatory mechanisms of DBD in the treatment of benzene-induced BDS using multi-omics techniques, providing molecular targets and pathway insights for its clinical application in BDS-related disorders. Furthermore, the therapeutic strategies highlighted—such as “DNA repair → cell cycle restart → erythroid differentiation” and “immune microenvironment remodeling”—offer novel perspectives for intervening in other hematopoietic suppression diseases or immune dysfunction syndromes.

## Conclusions

DBD has significant therapeutic effects on benzene-induced BDS. It effectively restores hematopoietic function; normalizes peripheral blood parameters; repairs pathological damage to the thymus, spleen, and bone marrow; and attenuates inflammatory responses. Mechanistically, our multi-omics data suggest that DBD likely acts through synergistic mechanisms involving hematopoietic lineage repair and immune microenvironment remodeling. Specifically, DBD promotes iron-dependent erythropoiesis by upregulating TFRC, downregulating KITLG and FLT3LG to coordinate stem cell pool protection and ensure sustainable hematopoiesis, and modulating ITGA4(↑)/ITGA1(↓) to increase stem cell homing, inhibit fibrosis, and preserve physiological hematopoietic niches. Additionally, DBD appears to enhance MHC class II antigen presentation and immune cell activation, thereby promoting immune homeostasis and modulating compensatory stress responses in BDS. These findings not only support the blood-replenishing function of this classic formula at the molecular level but also illustrate its holistic regulatory capacity in counteracting benzene-induced hematopoietic failure. The identified targets and pathways provide a mechanistic foundation for the clinical application of DBD and establish a translatable therapeutic paradigm for BDS and related hematopoietic–immune disorders.

## Supplementary Information

Additional file 1.

## Data Availability

No datasets were generated or analysed during the current study.

## Abbreviations

BDS: Blood deficiency syndrome
DBD: Danggui Buxue Decoction
TCM: Traditional Chinese medicine
rhEPO: Recombinant human erythropoietin
STZ: Stanozolol
RBC: Red blood cell
MCV: Mean corpuscular volume
MPV: Mean platelet volume
HCT: Hematocrit
PCT: Plateletcrit
TPO: Thrombopoietin
IL-1β: Interleukin-1β
EPO: Erythropoietin
G-CSF: Granulocyte colony-stimulating factor
IFN-γ: Interferon-γ
PCA: Principal component analysis
DIA: Data-independent acquisition
FC: Fold change
GO: Gene Ontology
KEGG: Kyoto Encyclopedia of Genes and Genomes
DEGs: Differentially expressed genes
TFRC: Transferrin receptor
KITLG: KIT ligand
FLT3LG: Fms-related tyrosine kinase 3 ligand
ITGA4: Integrin alpha 4
ITGA1: Integrin alpha 1
APCs: Antigen-presenting cells
HSCs: Hematopoietic stem cells
SDS-PAGE: SDS-polyacrylamide gel electrophoresis
LC-MS/MS: Liquid chromatography-tandem mass spectrometry
